# The Optimal Initial Dose and Route of Naloxone Administration for Successful Opioid Reversal: A Systematic Literature Review

**DOI:** 10.7759/cureus.52671

**Published:** 2024-01-21

**Authors:** Rida Aziz, Lan Nguyen, Washika Ruhani, An Nguyen, Brian Zachariah

**Affiliations:** 1 Pain Management, William Carey University College of Osteopathic Medicine, Hattiesburg, USA; 2 Emergency Medicine, William Carey University College of Osteopathic Medicine, Hattiesburg, USA

**Keywords:** opioid withdrawal, opioid pandemic, drug and substance abuse, initial stability, opioid use disorders, opioid reversal, buprenorphine and naloxone

## Abstract

This systematic literature review aims to determine the optimal initial dose of naloxone for successful opioid overdose reversal across different administration routes. Types of participants included adults who have opioid overdoses and adults who are suspected to have opioid overdoses. Pregnant women, children, animals, and populations outside the US were excluded. The interventions included were intranasal (IN), intramuscular (IM), and intravenous (IV) naloxone administration. The data collected for this systematic review were studies from PubMed, CINAHL, PsyINFO, and Cochrane Central Register of Controlled Trials registers between January 2015 and July 2021. The risk of bias was assessed via the Review Manager application. Six studies met the inclusion criteria. A meaningful statistical analysis was unable to be conducted with such few studies. The studies reveal 2 mg IN as the most popular dosing for initial naloxone for successful opioid reversal. The most common route of naloxone administration for successful reversal could not be studied but most studies revealed successful initial naloxone dosing in IN equivalents. With minimal studies emerging from our review, further research is warranted in naloxone dosing for optimal opioid reversal in order to fully treat patients. Healthcare workers must be vigilant of potential withdrawal from high naloxone dosing as well as the inefficiency of lower naloxone dosing for adequate opioid overdose reversal in order to treat patients with opioid overdoses properly.

## Introduction and background

For decades, opioid overdose and addiction have been an epidemic in the nation. Drug overdose is the leading cause of injury-related death in the United States [1]. This epidemic has only been worsening exponentially. Opioid overdose deaths doubled from 2010 to 2016: 21,089 in 2010 to 42,249 in 2016 [2]. The CDC reports that in 2017 there was a 10% rise in deaths related to drug overdose [1]. Furthermore, overdose deaths have only risen during the COVID-19 pandemic [1]. In 2021, one in 22 deaths in the United States were due to unintentional opioid overdose with a concerning one in 10 deaths in men ages 15-19 [2]. Patients who have had one overdose are more likely to suffer from additional overdoses. Opioid overdoses involve altered mental status, miosis, and most alarming, respiratory failure. As such, the opioid crisis has only risen in the past few years and must be a priority. Proper treatment for overdoses, which entails the proper initial naloxone dosing, is at the forefront of issues the medical community is tackling in order to save lives and address this opioid crisis [1,3].

Fortunately, naloxone, an opioid antagonist that is commonly known as Narcan, is a very effective treatment for opioid reversal for patients. Narcan normally has 4mg of naloxone given intranasally. The most common routes naloxone is given are intramuscular (IM), intranasal (IN), and intravenous (IV). However, in 2021 the FDA approved an 8 mg IN dosage of naloxone [4]. We are seeing a trend of increased dosing of naloxone being used. Furthermore, IN is not the only mode in which naloxone is being given. Naloxone can be given intranasally, intramuscularly, and intravenously. Each form that naloxone is given has different dosages at which naloxone works the most efficiently [4].

Naloxone, a synthetic counterpart of oxymorphone, acts as an opioid blocker that targets three specific opioid receptors which are μ, k, and σ, all of which are located in the brain [5]. This drug can quickly penetrate the brain and counteract opioids, boasting a distribution half-life of 4.7 minutes and an elimination half-life of 65 minutes [5]. Due to its brief half-life, there is a risk of patients experiencing a return of toxicity and breathing difficulties which is described as re-narconization. Nevertheless, how well naloxone works can vary from person to person. For instance, the CYP2D6 gene, which dictates the production of the P4502D6 hepatic enzyme, can differ in its expression among individuals [5]. This variance in genetic encoding influences how receptors bind and function.

Several studies on animals and humans show that a dose-dependent response exists when it comes to naloxone’s efficacy in reversing factors such as respiratory depression [5]. Since resistance to naloxone is shown through studies done on both populations, higher doses of naloxone for opioid overdose reversal are deemed necessary to exceed the threshold of fentanyl at the μ/mu-opioid receptors in the central nervous system. Certain studies highlight that the common practice of redosing naloxone in two-minute intervals with the current dosage is not sufficient for a rapid reversal, especially when a non-professional is administering [6]. Nevertheless, other studies show that the effects of high-dose naloxone are significant including the release of catecholamines which can cause cardiac arrhythmias and pulmonary edema or even mild behavioral disturbances [6]. To minimize these adverse effects, certain studies have recommended using the lowest dose of naloxone.

Since such controversy still surrounds what the effective dosage of naloxone should be, more research is needed to identify whether an increase is truly necessary and what that dosage is to prevent harmful outcomes. The purpose of this literature review is to identify the optimal dosing of naloxone to help opioid overdose reversal and highlight the lack of literature that still exists on this issue.

## Review

Methods

Inclusion Criteria

Types of participants include adults who have opioid overdoses and adults who are suspected to have opioid overdoses. The interventions included in the search were IN, IM, and IV naloxone administration. The studies looked at were retrospective cohort study, retrospective cross-sectional study, morbidity and mortality report, and prospective interventional study. The settings looked at were Emergency Department, EMS, EMT, in-patient, and outpatient. 

Exclusion Criteria

Exclusion criteria include pregnant women, children, animals, and populations outside of the US as we wanted to focus our studies on optimal results with a decreased amount of variability in our results. As naloxone dosing will vary among non-pregnant adults, pregnant women, and children and skew results, we focused on non-pregnant adults. Furthermore, we wanted to focus on patients in the United States, especially in regard to the opioid crisis in the nation. Opioid overdoses can be due to a mixture of different synthetic and non-synthetic opioids and different nations have different opioids that are currently popular recreationally and regionally. As such, we focused on studies in the United States. Case studies were also excluded since these studies only focused on one/a few cases. Specific dosages (such as 8 mg instead of high dose) were not used within the selection process so as to not favor certain dosages over others, as well as to allow us a broader catch of articles. 

Information Sources

All of the searches were electronic searches; no printed texts were used. The databases used were PubMed, CINAHL, PsyINFO, and Cochrane Central Register of Controlled Trials registers between January 2015 and July 2021.

Selection Process

Keywords used included naloxone; systematic review; opioid reversal; opioid overdose; intramuscular; intravenous; intranasal; initial dosage; pharmacokinetics; in-patient; out-patient; emergency room; first responders; EMS. Our search strategy consisted of the following search terms: “(Naloxone initial dosage OR naloxone initial dose OR narcan initial dosage OR narcan initial dose) AND (opioid reversal OR opioid overdose)” which resulted in four studies. “(Naloxone OR narcan) AND (initial dosage OR initial dose) AND (opioid overdose OR opioid reversal)” which resulted in four studies. “(Intramuscular OR IM) AND (naloxone OR narcan) AND (opioid overdose OR opioid reversal) AND (initial dosage OR initial dose)” which resulted in 11,774 studies. “(Intravenous OR IV) AND (naloxone OR narcan) AND (opioid overdose OR opioid reversal) AND (initial dosage OR initial dose)” which resulted in 52 studies. “(Intranasal OR IN) AND (naloxone OR narcan) AND (opioid overdose OR opioid reversal) AND (initial dosage or initial dose)” which resulted in two studies. “(Naloxone OR narcan) AND (opioid overdose OR opioid reversal) AND (initial dose OR initial dosage) AND (in-patient OR inpatient)” which resulted in three studies. “(Naloxone OR narcan) AND (opioid overdose OR opioid reversal) AND (initial dose OR initial dosage) AND (out-patient OR outpatient)” which resulted in 57 studies. “(Naloxone OR narcan) AND (opioid overdose OR opioid reversal) AND (initial dose OR initial dosage) AND (emergency room OR emergency department OR ER OR ED)” which resulted in one study. “(Naloxone OR narcan) AND (opioid overdose OR opioid reversal) AND (initial dose OR initial dosage) AND (EMS OR EMT) NOT (first responders)” which resulted in one study. “(Naloxone OR narcan) AND (opioid overdose OR opioid reversal) AND (initial dose OR initial dosage) AND (pharmacokinetics)” which resulted in 57 studies.

Outcomes of Interest

Outcome measures were the initial dosage of naloxone used for successful reversal and the route of administration. Since this SLR aimed to determine the most appropriate initial dose of naloxone in response to opioid overdose across different administration routes, such as IN, IM, and IV, we standardized the unit of analysis across the six chosen studies as the success of opioid reversal by the improvement of respiratory rate after one dose of naloxone. The reversal of opioid overdose was defined as an improvement of respiratory depression and altered mental status that would not require additional doses of naloxone after initial naloxone administration.

We did not take into account the need for an additional total dose of naloxone after an initial dose required for improvement of respiratory rate. As for subgroup analysis and investigation of heterogeneity, there are none because these studies involve adult patients with symptoms of suspected opioid overdose. Our SLR does not subcategorize these adults into subgroups of different types of opioid use. No secondary outcomes were analyzed. 

Statistical Analysis

Due to the low volume of studies, a meta-analysis was unable to be conducted in this review.

Risk of Bias Assessment

The risk of bias was calculated via the Review Manager application.

Results

Study Selection

**Figure 1:**
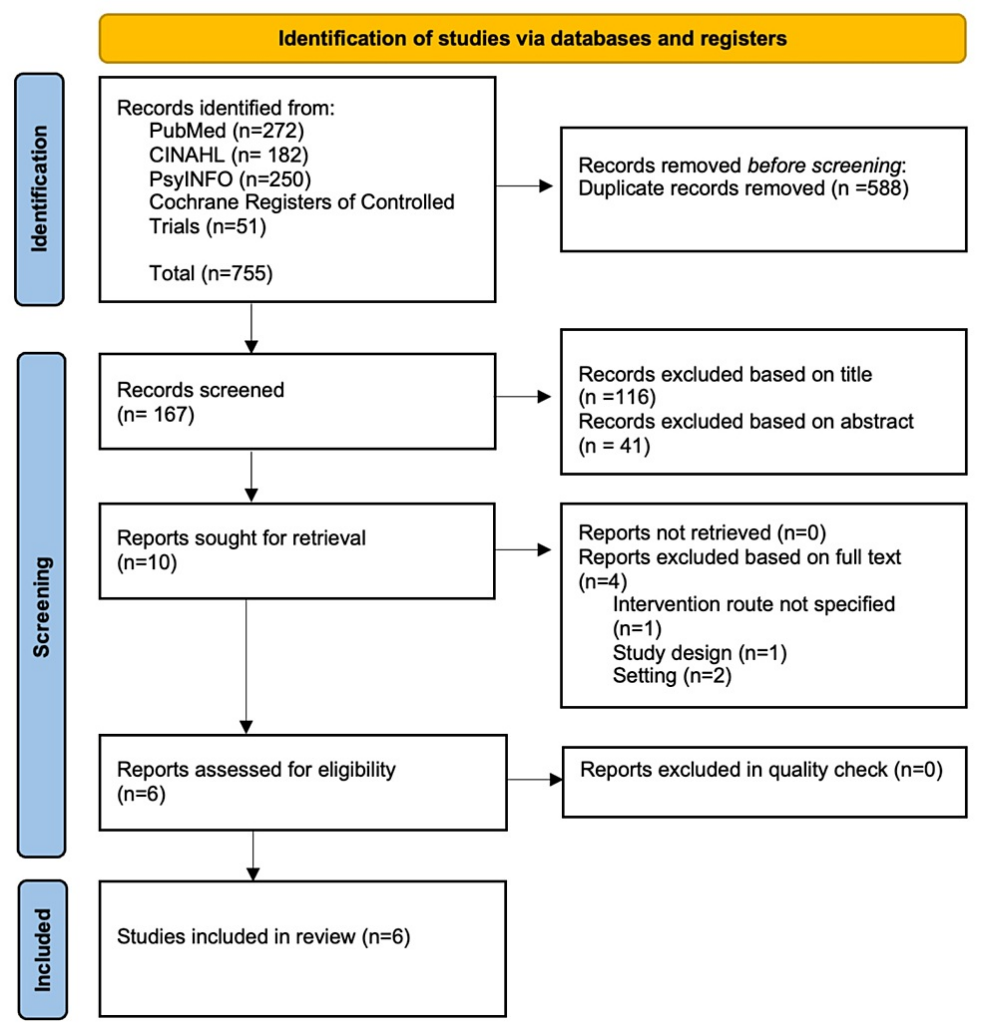
PRISMA Flowchart Preferred Reporting Items for Systematic Reviews and Meta-Analyses (PRISMA) 2020 flowchart illuminating our study selection [7]

A total of 755 studies were collected from PubMed, CINAHL, PsyINFO, and Cochrane Central Register of Controlled Trials registers between January 2015 and July 2021. Our systematic literature review was registered and published on the PROSPERO website on August 27, 2021. From that point, we began excluding studies, as depicted in the flow diagram in Figure 1.

After non-relevant and duplicated studies were removed, 51 were left. Of those, we screened the studies once again and excluded the ones that did not fit our above criteria. The criteria that most of these studies did not fit include: the outcome not measuring the number of opioid reversals following initial naloxone administration, the study design not fitting our criteria (randomized controlled trials, observational studies, and cohort studies), the setting of the study being outside of the United States of America, the intervention not specifying initial dosage of naloxone, and the population not being restricted to adults. Next full-text articles were assessed, and 10 studies were excluded due to similar reasonings (setting, study design, and intervention route). By the end of the selection process, six studies were left that fit our criteria. Overall, data were extracted by noting the dosage related to successful opioid reversals from the studies. The treatment effect was measured in successful opioid overdose reversal, with the treatment indicating the initial dosage of naloxone via IM, IN, or IV (excluding oral and cutaneous routes). The success of initial doses of naloxone is demonstrated as the reversal of respiratory rate depression.

Study Characteristics/Outcomes

With the selection criteria mentioned above, six studies are included in this review. The six studies are summarized in Table 1. 

**Table 1:** Summary of six studies

Study	Summary
Tomassoni AJ, et al. [8]	Cohort of 12 adults with fentanyl overdose at Yale New Haven Hospital, New Haven, Connecticut on June 23, 2016. 9 patients were admitted to the hospital, including 4 to the intensive care unit (ICU) and 3 died. 1 out of total 12 patients with confirmed positive urinary analysis of fentanyl overdose received one initial dose of 2 mg Naloxone intranasally was discharged. Of note, both IN and/or IV/Intraosseous injection were used by EMS. Most patients in this study had both routes of naloxone by EMS.
Rando J, et al. [9]	A Prospective Study in Lorain County, OH with 247 individuals. Trained police officers in Lorain County, Ohio were trained to administer an IN 2 mg naloxone kit on suspected opioid overdose victims before EMS and successfully revived 52 out of 67 patients.
Klebacher R, et al. [10]	Retrospective chart review of Electronic Medical Records (EMR) at Newark Beth Israel Medical Center of 2166 adults with suspected opioid overdose that were treated with naloxone by EMS. The EMS service in New Jersey recorded 1971 out of 2166 suspected opioid overdose victims who survived with 2 mg of IN, with no redosing needed.
Thompson J, et al. [11]	Retrospective, cross-sectional study of 218 adult patients with pre-hospital opioid overdose at EMS in 2 neighboring counties of Southeast Michigan: Oakland County and Washtenaw County. 94 pts in Oakland county and 124 pts in Washtenaw county. 54 out of 124 adult patients of Washtenaw county, MI reported by chart review as a positive response to initial 1.77 average mg IN naloxone reversal. Also, 39 out of 94 adult patients of Oakland county, MI reported by chart reviews as a positive response to initial average 0.48 mg IN naloxone reversal. Intranasal naloxone at an initial dose of 0.48 mg was equally effective during the prehospital period as treatment at an initial dose of 1.77 mg, was associated with a lower rate of adverse effects, and represented a 79% lower cost.
Wong F, et al. [12]	Retrospective cohort study of 84 adults (18+) who present to Emergency Department (ED) with opioid intoxication and received IV Naloxone at 2 different EDs in the same urban city (location not disclosed). Study showed that the time to recurrence of toxicity after opioid overdose in the ED was not dependent on the first dose of IV naloxone administered (0.4mg IV vs 1-2 mg IV). Additionally, it shows that 18 out of 42 patients with opioid overdose were successfully reversed with 0.4 mg IV without the need of redosing additional doses of naloxone.
Carpenter J, et al. [13]	A retrospective chart review of 837 charts, with 121 subjects at a single emergency department and its affiliated emergency medical services (EMS) agency in Atlanta. IV was the most common route, given to 62% of subjects, IN given to 38%, IM given to 25% and interosseous (IO) given to 1%. Naloxone was shown to be successful in overdose reversal in 93% of cases, and only 11% precipitating withdrawal. The median dose of naloxone for patients with precipitated withdrawal was 0.6 mg IV. The median dose of naloxone for patients successfully treated with naloxone were given an initial dosage of 0.8 mg IV.

Furthermore, these studies were then analyzed based on successful initial naloxone dosing as well as the route taken (IM, IV, IN) as depicted in Table 2. As shown in the table, the IN route was most commonly used initially pre-admission to ED by EMS. 

**Table 2:** Successful initial doses and routes (IM, IV, IN) of naloxone for the six studies are observed

Initial Dosage and Route	Study with the Outcome
2 mg IN Naloxone	Tomassoni AJ, et al. [8]
2 mg IN Naloxone	Rando J, et al. [9]
2 mg IN Naloxone	Klebacher R, et al. [10]
1.77 mg IN and 0.48 mg IN Naloxone	Thompson J, et al. [11]
0.4 mg IV Naloxone	Wong F, et al. [12]
0.8 mg IV Naloxone	Carpenter J, et al. [13]

Successful outcomes are defined as reversal of respiratory rate depression and/ or improvement of altered mental status without the need for another second dose of naloxone. Moreover, the result of the high dose (1 mg-2 mg) group of the article Wong et al. is not included since the article had a small sample size for "high dose" and could not specify a certain dosage [12].

Risk of Bias in Studies

**Figure 2:**
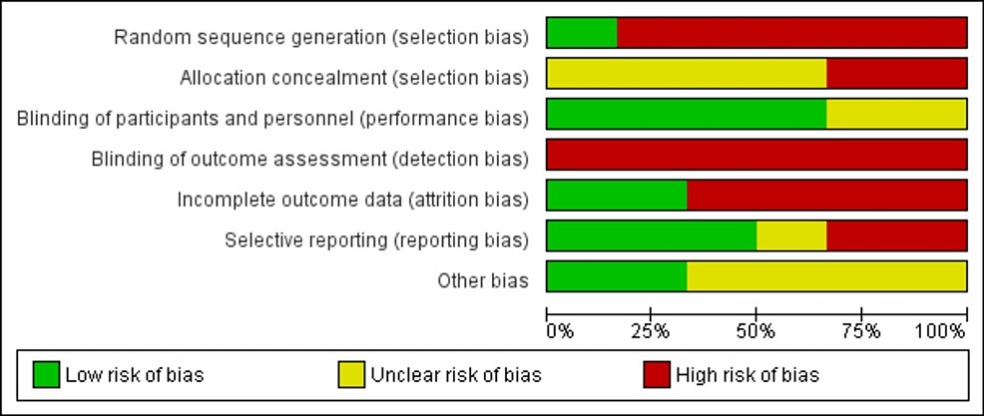
Risk of bias graph: our judgments about each risk of bias item presented as percentages across all included studies

**Figure 3:**
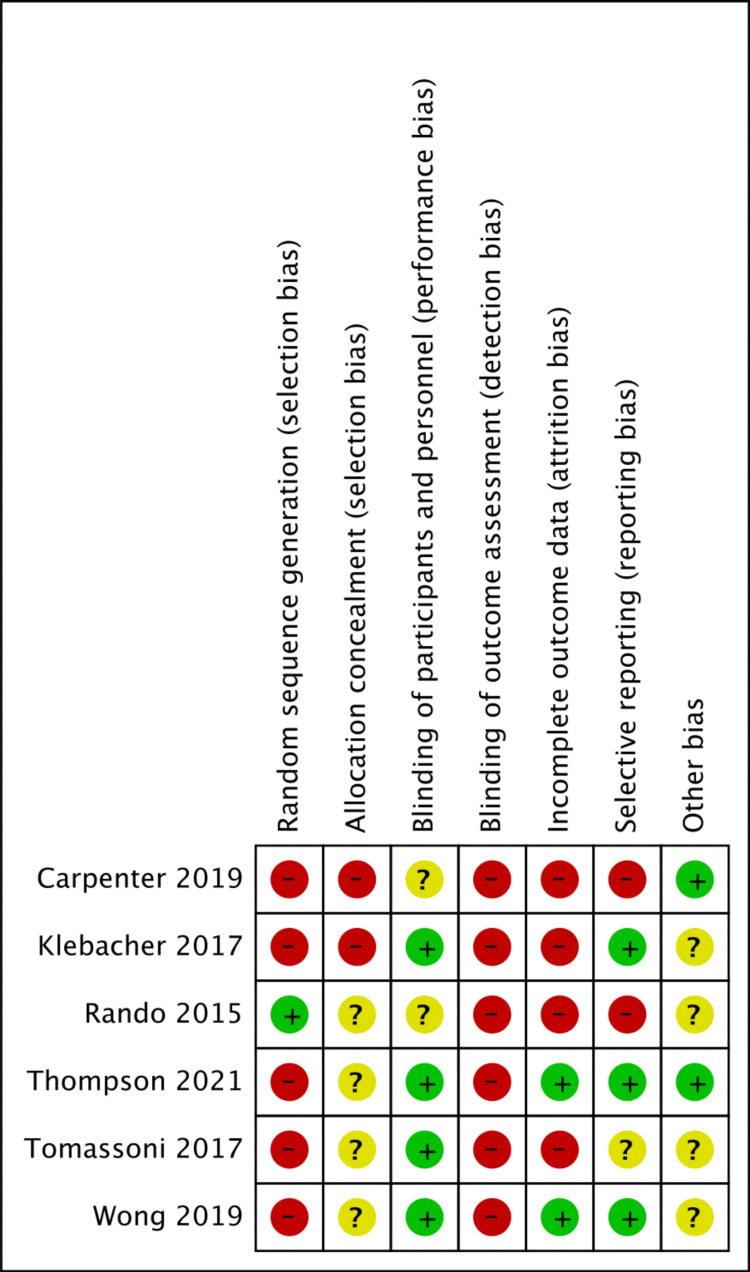
Risk of bias summary: our judgments about each risk of bias item for each included study Bias of all six studies [8-13]

Figure 2 depicts the likelihood of each type of bias throughout the studies. From that, it is apparent detection bias is the highest risk and performance bias is the lowest. Figure 3, in contrast, depicts the risks of bias for each individual study. From that, it is apparent that Carpenter 2019 had the most risk of bias and Thompson 2021 had the least risk of bias. 

Discussion

This SLR aimed to investigate an appropriate initial dose of naloxone across multiple routes (IM, IN, and IV) that successfully reversed opioid overdose. The reversal of opioid overdose was defined as an improvement of respiratory depression and altered mental status that would not require additional doses of naloxone after initial naloxone administration. By reviewing 755 articles in our SLR, six studies were selected according to search criteria as described in the method. Due to the small number of included studies, there was no meta-analysis conducted. Although there is no standard initial dose of naloxone in IM, IN, and IV routes, the initial doses used in these studies demonstrated the possibility of success in reversing respiratory depression and altered mental status without the need to administer more doses. However, our screening yielded no papers with results of dosing IM routes. Interestingly, Carpenter included the IM route in its study [13]. However, for the sake of continuity, the dosages presented in the results of that study were all calculated to their IV equivalents. As such, dosing for IM naloxone was not adequately studied in our results. 

In particular, the study reveals 2 mg IN as the most common initial dosage among the studies of naloxone for the reversal of opioid overdose. Three of the studies showed 2 mg IN as the dosage used for successful reversal of opioid reversal. Only one study showed success with 0.8 mg IV, one study showed success with 0.4 mg IV, and one study showed success with 1.77 mg IN and 0.48 mg IN. This is interesting because although the FDA has been shown approving higher doses of naloxone (4 mg IN and 8 mg IN), our study shows 2 mg IN as the most successful initial dosage of naloxone for opioid reversal [4]. In fact, none of our studies shows data for 4 mg and 8 mg IN of naloxone. 

Furthermore, our SLR shows IN as the most common route for naloxone. Four out of the six studies show success with IN naloxone (including all the studies showing success with a 2 mg dosage), two studies show success with IV and no studies included that gave IM dosing for naloxone.

Implications

The discrepancies between our studies can be due to variations in patient populations, as some are in more urban areas while others were in rural places, and the type of opioid the patients were ingesting, as some of them could have been mixed with synthetic opioids such as fentanyl. These are all challenges that healthcare workers face when combating the opioid crisis. Without a standard optimum reversal, we cannot ascertain an effective reversal and open patients up to adverse effects which can include both continued respiratory failure or withdrawals. Furthermore, with Carpenter showing their results of all naloxone given in IV equivalents, no data was given with IM dosing of naloxone. As such, we must question the validity of the most common route of naloxone due to the unequal representation in our articles. 

The discussion about whether to increase the initial dosage of naloxone remains controversial. Studies that encourage a higher initial dosage of naloxone reveal its safety and rapid reversal of withdrawal symptoms. For example, a case report studying fentanyl-laced drugs found that partial opioid agonists such as buprenorphine can displace full opioid agonists of the mu receptors causing withdrawal but this can be reversed with a high dose by oversaturating mu receptors with buprenorphine [14]. Another study analyzing a survey given to US residents who used 4 mg of Narcan to reverse opioid overdose on others found that these participants preferred and had more confidence in the use of 8 mg of Narcan, which is an even higher dose as a bystander [15]. Furthermore, a retrospective cohort study revealed that even though individuals treated with initial high-dose naloxone were more likely to have opioid withdrawal, they were still more likely to have opioid reversal compared to those who received low-dose naloxone. Their study also showed that those receiving low doses of naloxone needed re-dosing post-reversal more often than those receiving high-dose naloxone [16]. However, there are also studies cautioning on the effects of high-dose naloxone. High doses of naloxone can increase the release of catecholamines, leading to cardiac arrhythmias and pulmonary edema [17,18]. Although the routes were not mentioned, the administration of 4.25 mg of naloxone causes severe pulmonary edema [18]. As a result, patients were on mechanical ventilation for at least two days, with one case requiring venovenous extracorporeal membrane oxygenation support [18]. In addition, naloxone induces a wide range of acute withdrawal symptoms. Among the common acute withdrawal symptoms are nausea and vomiting, which can lead to aspiration, as well as pulmonary edema, arrhythmias, cardiac arrest, and blood pressure instability [6,17]. Thus, increasing initial doses of naloxone is met with resistance due to the potential for adverse events of arrhythmias, respiratory distress, and withdrawal symptoms. 

Current literature expands on available routes of administration, such as buccal and sublingual, which allow patients greater access to naloxone [19]. Additional studies researching treatment protocols can be helpful since there is a lack of studies currently discussing the topic of increasing the dose of naloxone for rapid opioid reversal. Since these limitations hinder the overall completeness and applicability of evidence, further research is warranted to improve our model.

There are multiple studies in the literature that investigated the relationship between doses of naloxone and the naloxone adverse effects such as nausea, pulmonary edema, arrhythmia, and vomiting. In fact, in healthy adult volunteers, higher doses of IV naloxone at 2-4 mg/kg showed a dose-dependent relationship in increasing both systolic pressure and respiratory rate [20]. More cases of tachycardia were observed in patients who were administered the naloxone standard dose of 0.4-2 mg IV compared to 0 cases of tachycardia in patients with the initial naloxone doses of < 0.4 mg IV in one SLR of naloxone dosing in the ED [21]. These adverse effects must be taken into account with higher naloxone dosing. Furthermore, another study showed that the reversal of synthetic opioids fentanyl is not dependent on increasing doses of naloxone [13]. Currently, there are no established standard initial doses for administering naloxone, the FDA recently approved the 8 mg IN spray in 2021 and recommended that bystanders administer any available doses of naloxone on hand [4]. With an inconclusive agreement on the initial naloxone doses and the need to administer additional doses, healthcare workers must be cognizant that the half-life of naloxone in adults can vary between 30 and 80 minutes [22].

Limitations

This SLR consists of certain limitations. Specifically, the review was narrowed down from 755 to only six studies after defining the search criteria and following the method for search strategy. One case study report was excluded due to the sample size of one. Moreover, a group in another study was not used because it failed to specify whether an individual group was either 1 mg or 2 mg IV naloxone but instead grouped the two doses as one homogenous group and labeled it as “high dose IV naloxone.” In our initial screening among the databases, for initial naloxone dosage of opioid overdose in the ER, we only received one study. For inpatient, we received three studies and for outpatient, we received 57 studies. Furthermore, it was important to look for the initial dosage of naloxone for our study, which excluded many studies that did not report initial dosing and/or saw no success with initial dosing. Shifting the view of our study to the initial dose was crucial for our study, as we wanted to define an optimal initial dose for naloxone overdoses with successful reversal, not secondary/additional doses given that cause successful reversal. These limitations excluded many studies that gave us a limited view on naloxone dosing for opioid reversal. As such, conclusions made from our results must take these limitations and limited views into account. 

Another limitation includes the population that was studied. The studies in this review have their population limited to only adults in the US who have been exposed to opioids. With this being said, other relevant populations, such as pregnant individuals and children were excluded. Besides, the studies were published between January 2015 and July 2021. This poses a problem as newer publications that are not included in the review could provide more insights about the topic. Further limitations of this SLR include not being able to quantitatively analyze the evidence using a meta-analysis. This occurred because of the few studies that were remaining for analysis. The statistical process of analysis and combining results could not be achieved through this study. As such, any conclusions made from this study must take into account the limited scope and scarcity of articles used.

In addition, this SLR has limited quality of evidence as a consequence of the articles that were pulled which had limitations of their own. A major limitation was the selection bias, mainly retrospective cohort studies. Furthermore, small sample sizes in the studies increase the chances of a type II error**. **Wong et al. note a lack of documentation for recurrences of opioid toxicity and had to rely on measurements of repeat given naloxone for this assumption [12]. A lack of standardization of protocol for secondary and repeat doses of naloxone was also illustrated [12]. Consequently, it was left to the current physician’s input leading to variability. Additionally, the studies of Wong et al., Carpenter et al., and Tomassoni et al. highlight that the detectionof semi-synthetic and synthetic opioids, such as fentanyl, as well as their metabolites, in the urine drug screening was not reliable [8,12,13]. Hence, relations between the type(s) of opioids and their effect on naloxone dosing could not be observed. Unknown confounding factors can affect the significance and reliability of these results. Incomplete documentation for cases also played a role. For instance, it is mentioned in Thompson et al. thatthe authors had access to only the prehospital records but not the inpatient ones, creating inherent biases in the results [11]. In Klebacher et al., they were unaware if first responders had given naloxone prior to ED [10]. As such the true initial dosage** **of naloxone, as well as the potential reversal from the initial dosage, may not be the one documented in the studies [10]. All of these limitations have the potential to alter outcomes measured within this study: the true initial dosage of naloxone administration that leads to successful reversal.

Finally, there are several biases in this review, including reporting bias of how positive findings are more commonly published than negative findings. Moreover, in Carpenter et al., the abstractors were not blinded to the study hypothesis [13]. This could affect the response to “based on your review of the ED chart, you believe that naloxone improved the patient’s clinical status,” as this is a subjective question [13]. In the same article, the study obtained a urine drug screen (UDS) [13]. This could have been because the presentation or clinical course of patients deviated from a perceived norm. Cases in the included subjects with a UDS did differ from those excluded since no UDS was obtained could pose a potential source of bias. Additionally, the subjects in this study had an Against Medical Advice (AMA) rate of 4%. This is low compared to the AMA rate of subjects in other studies. For instance, Scheuermeyer et al. revealed that 10.4% of patients left immediately, and later an additional 14.3% left AMA [23]. This difference in the AMA rates could be due to the severe clinical conditions of patients in Carpenter et al. that also allowed their UDS to be obtained [13]. This may have biased the results toward more severely overdosed patients, leading to an overestimation of the naloxone dose required. In Klebacher et al., among the 195 patients who required further intervention, paramedics preferred to administer naloxone intravenously, rather than a second IN administration after a failure to improve after the initial IN administration [10]. This preference may have biased their providers toward the IV route. 

## Conclusions

There is a critical need for further research to establish standardized initial doses of naloxone for effective opioid overdose reversal. Additionally, with increasingly high dosing of naloxone being approved (such as 8 mg IN), the success rate of 2 mg IN naloxone in our studies for opioid reversal question the need and practicality of such high dosing of naloxone for successful opioid reversal. This is necessary clinically as the opioid crisis is only worsening post-COVID-19 in the United States. As healthcare workers, it is vital that we can reverse the life-threatening effects of opioid overdose quickly and consistently without placing patients into excessive withdrawals.
